# Preparation of a Single-Chain Antibody against Nucleocapsid Protein of Porcine Deltacoronavirus by Phage Display Technology

**DOI:** 10.3390/v14040772

**Published:** 2022-04-08

**Authors:** Yixuan Zhang, Yue Song, Haojie Ren, Quan Zeng, Yixin Yuan, Lu Xia, Zhanyong Wei

**Affiliations:** 1 College of Veterinary Medicine, Henan Agricultural University, Zhengzhou 450046, China; zhangyixuan319@gmail.com (Y.Z.); songyue2008@163.com (Y.S.); renhaojie1995@126.com (H.R.); zq779196981@163.com (Q.Z.); yuanyixin94@163.com (Y.Y.); 2 Molecule Biology Laboratory, Zhengzhou Normal University, Zhengzhou 450044, China; 3 Key Laboratory for Animal-Derived Food Safety of Henan Province, Henan Agricultural University, Zhengzhou 450046, China

**Keywords:** porcine deltacoronavirus (PDCoV), N protein, phage display technology, single-chain fragment variable (scFv), antigen epitope

## Abstract

Porcine deltacoronavirus (PDCoV) mainly causes severe diarrhea and intestinal pathological damage in piglets and poses a serious threat to pig farms. Currently, no effective reagents or vaccines are available to control PDCoV infection. Single-chain fragment variable (scFv) antibodies can effectively inhibit virus infection and may be a potential therapeutic reagent for PDCoV treatment. In this study, a porcine phage display antibody library from the peripheral blood lymphocytes of piglets infected with PDCoV was constructed and used to select PDCoV-specific scFv. The library was screened with four rounds of biopanning using the PDCoV N protein, and the colony with the highest affinity to the PDCoV N protein was obtained (namely, N53). Then, the N53-scFv gene fragment was cloned into plasmid pFUSE-hIgG-Fc2 and expressed in HEK-293T cells. The scFv-Fc antibody N53 (namely, scFv N53) was purified using Protein A-sepharose. The reactive activity of the purified antibody with the PDCoV N protein was confirmed by indirect enzyme-linked immunosorbent assay (ELISA), western blot and indirect immunofluorescence assay (IFA). Finally, the antigenic epitopes that the scFv N53 recognized were identified by a series of truncated PDCoV N proteins. The amino acid residues ^82^GELPPNDTPATTRVT^96^ of the PDCoV N protein were verified as the minimal epitope that can be recognized by the scFv-Fc antibody N53. In addition, the interaction between the scFv-Fc antibody N53 and the PDCoV N protein was further analyzed by molecule docking. In conclusion, our research provides some references for the treatment and prevention of PDCoV.

## 1. Introduction

Porcine deltacoronavirus (PDCoV) is an enteropathogen that can infect pigs of all ages. The common clinical symptoms of PDCoV disease are dehydration, acute diarrhea and vomiting [1,2]. The mortality rate of PDCoV-infected piglets is as high as 20–70% [3,4]. PDCoV was first detected in clinical samples of pigs in Hong Kong in 2012 and was first successfully isolated in diarrheic piglets in the United States in 2014 [4,5,6]. Since then, PDCoV has been detected in clinical samples of diarrheic pigs in many countries and regions, which seriously endangers the development of the pig industry throughout the world. Moreover, PDCoV commonly co-infected with porcine epidemic diarrhea virus (PEDV) and transmissible gastroenteritis virus (TGEV) in natural infections. The clinical symptoms of PEDV, TGEV and PDCoV, which lead to increased mortality in piglets, are similar. An early study showed that PDCoV could bind to human APN, which suggested a potential risk of human infection. Furthermore, this conclusion was supported by a recent report that three young children in Haiti were infected with PDCoV [7,8].

PDCoV is an enveloped, single-stranded RNA virus belonging to the *Deltacoronavirus* genus in the family *Coronaviridae* [9]. Its genome is about 25 kb in size and is arranged in the following order: 5’ untranslated region (UTR), open reading frame 1a/1b (ORF1a/1b), spike protein (S), envelope protein (E), membrane protein (M), nonstructural protein 6 (NS6), nucleocapsid protein (N), nonstructural protein 7 (NS7) and 3’ UTR [6,10]. Among them, the N protein is highly conserved in the coronavirus genus and is one of the most abundant structural proteins in virus-infected cells [11]. The N protein is located inside the virus particle and packages the viral RNA genome into a long helical ribonucleoprotein complex. The N protein plays essential roles in viral life cycles, involved in viral RNA synthesis and the regulation of cellular processes. Moreover, the N protein is also an immunodominant antigen in host immune responses that can be used as a diagnostic antigen and immunogen [12,13].

Single-chain variable fragment (scFv), also called single-chain antibody, is one of the genetically engineered antibodies. It is a single polypeptide chain that is composed of a heavy-chain variable region (V_H_) and a light-chain variable region (V_L_) through a flexible polypeptide (linker) about 50–90 bp [14]. ScFv can spontaneously fold into antigen-binding variable fragment (F_V_) segments and increases the stability of F_V_. ScFv is one of the most frequently reported small molecular antibodies and is more conveniently constructed and expressed in vitro compared to traditional monoclonal antibodies (mAbs) [15,16]. Currently, scFv is being widely applied in the diagnosis and treatment of virus infections [17,18].

In this study, a porcine phage display scFv library was constructed, and phage display technology was used to obtain specific scFv targeting the PDCoV N protein. The positive scFv colony N53 was identified and expressed in HEK-293T cells. Then, the reactivity and specificity of this scFv was evaluated. Finally, the antigen epitope that the scFv-Fc antibody N53 recognized was confirmed. This study may be helpful for the prevention and treatment of PDCoV infection.

## 2. Materials and Methods

### 2.1. Bacteria Strains, Plasmids, Cell Lines and Viruses

*E. coli* XL1-Blue, pSEX81 phage display vector and pFUSE-hIgG-Fc2 vector were kindly provided by Molecule Biology Labarotory of Zhengzhou Normal University. pET-28a plasmid, pET-32a(+) plasmid, *E. coli* BL21 (DE3) and DH5α were preserved by our laboratory. M13KO7(ΔpIII) Hyper phage was purchased from PROGEN (PROGEN, Heidelberg, Germany).

Swine testis (ST) cells, Vero cells, and HEK-293T cells were cultured in Dulbecco’s Modified Eagle’s Medium (DMEM, Gibco, Carlsbad, CA, USA) containing 10% fetal bovine serum (FBS, Gibco, Carlsbad, CA, USA) at 37 °C with a humidified 5% CO_2_ incubator. PDCoV HNZK-02 strain, TGEV HN-2012 strain, PEDV CV777 strain and porcine sapelovirus (PSV) HNHB-01 strain were preserved by our laboratory. Viruses were propagated and identified according to our previous reports [19,20].

### 2.2. Antibody Variable Region Amplification and Full-Length scFv Assembly

Four 14-day-old diarrheic piglets that were RT-PCR positive for PDCoV but negative for PEDV, TGEV, porcine circovirus type 2 (PCV-2), pseudorabies virus (PRV) and porcine reproductive and respiratory syndrome virus (PRRSV) were selected [21]. The spleens of the four PDCoV-infected piglets were collected and ground together on ice. Total RNA was extracted from the spleen tissues using a TRIzol reagent (TransGen, Beijing, China). The complementary DNA (cDNA) was synthesized using a HiScriptⅡ1st Strand cDNA Synthesis Kit (Vazyme, Nanjing, China) according to the manufacturer’s instructions. Specific primers for antibody variable regions were designed according to the porcine antibody sequences published on NCBI (GenBank accession numbers: MK986903-MK986940; AF334738-AF334742; OK393047-OK393055). The primer sequences are listed in Table 1. V_H_ and V_L_ genes were separately amplified using sixteen pairs of primers using 2 × Taq Master Mix (Vazyme, Nanjing, China). The primer sequences are listed in Table 1. Briefly, nine pairs of primers were used to amplify the V_H_ gene (three forward primers: VH-1F, VH-2F and VH-3F; three reverse primers: VH-1R, VH-2R and VH-3R; cross-combination of the three forward primers and three reverse primers); six pairs of primers were used to amplify the V_L_κ gene (three forward primers: VLκ-1F, VLκ-2F and VLκ-3F; two reverse primers: VLκ-1R, and VLκ-2R; cross-combination of the three forward primers and two reverse primers); and one pair of primers was used to amplify V_Lλ_ gene (VL_λ_-1F and VL_λ_-1R). Then, all genes were purified via gel extraction, respectively. The purified V_H_ and V_L_ fragments were used as templates and spliced into scFv using the splicing by overlap extension PCR method (SOE-PCR). For SOE-PCR amplification, 40 ng of variable fragments (V_H_ and V_L_) and 12 μL of 2 × Taq Master Mix were mixed. The following program was used: 95 °C for 30 s, 56 °C for 30 s and 72 °C for 30 s, repeated for 20 cycles. After that, 1 μL of scFv-F and scFv-R primers was added, and the mixture was amplified for another 20 cycles as above. The scFv gene products were purified via gel extraction.

### 2.3. Construction of the Phage Display scFv Library

The purified scFv gene products and pSEX-81 phagemid vector were digested by *Sfi*I (NEB, Ipswich, MA, USA) and ligated by T_4_ DNA ligase (NEB, Ipswich, USA). The ligated product was desalted using the PCR Purification Recovery Kit (Qiagen, Hilden, Germany) and electroporated into *E. coli* XL1-Blue electro-competent cells to construct the antibody library. Then, 100 μL of transformant solution with a gradient dilution was coated on a 2 × YT agar plate contlines for Experimental Animaaining 100 μg/mL ampicillin and 1% glucose (2 × YT-AG), and the plate was incubated overnight at 37 °C. The library storage capacity was calculated using colony numbers in the diluted solution. Twelve colonies were randomly selected for identification by colony PCR using primers of pSEX81 as follows: 5′-TTCCGGCTCGTATGTTGTGT-3′ (pSEX81-F) and 5′-ACAACGCCTGTAGCATTCCA-3′ (pSEX81-R). The remaining transformant solution was cultured on a 2 × YT-AG plate overnight at 37 °C, and all the colonies were harvested and resuspended in 20 mL of 2 × YT-A medium containing 20% (*w*/*v*) glycerol. The resuspended cultures were stored at −80 °C as the bacteria library of the scFv gene. For the phage antibody library construction, the bacterial suspension above was incubated in 40 mL of 2 × YT-A medium at 37 °C until the optical density of the medium reached 0.5 (the absorbance at 600 nm, OD_600_ = 0.5). Then, M13KO7 helper phage with a multiplicity of infection (MOI) of 20 was added and incubated at 37 °C for 1 h. Bacteria were collected by centrifugation, further resuspended in 80 mL 2 × YT medium containing 100 μg/mL ampicillin and 50 μg/mL kanamycin (2 × YT-AK), and cultured overnight at 30 °C with shaking at 260 rpm. Phages were harvested from the culture supernatant by precipitation with 5 × PEG-NaCl (PEG 8000 (20%, *w*/*v*), 2.5 M NaCl) and resuspended in 1 mL of phosphate-buffered saline (PBS). One μL of the phage solution was incubated with 1 mL *E. coli* XL1-Blue cultures (OD_600_ = 0.5) at 37 °C for 1 h. The bacteria were gradient-diluted and coated on 2 × YT-AG plates for phage titer calculation.

### 2.4. Expression of the Complete and Truncated PDCoV N Protein

Recombinant plasmid pET-28a-PDCoV-N was constructed and used to express the PDCoV N protein. Briefly, viral RNA was extracted from the PDCoV CH-01 strain (Genbank accession number: KX443143.2), and cDNA was generated. The cDNA products were used as PCR templates with primers containing *Eco*RI and *Xho*I restriction enzyme sites to obtain the complete PDCoV N gene (Table 2); then, the N gene was cloned into plasmid pET-28a to obtain a pET-28a-PDCoV-N recombinant plasmid. The recombinant plasmid was transformed into *E. coli* BL21 (DE3) and grown in LB medium containing 100 μg/mL ampicillin (LB-A) at 37 °C. Isopropyl β-D-1-thiogalactopyranoside (IPTG) at a final concentration of 0.8 mM was added to the cultures when OD_600_ = 0.6, then cultured at 37 °C for another 6 h to induce protein expression. Bacteria were collected by centrifugation and resuspended in phosphate-buffered saline (PBS) and lysed on ice for sonication. After centrifugation, the recombinant protein in the supernatant was purified using Ni-Agarose His Purification Kit (CWBIO, Taizhou, China) according to the manufacturer’s instructions. The expressed protein was analyzed by sodium dodecyl sulfate-polyacrylamide gel electrophoresis (SDS-PAGE).

To express the truncated PDCoV N proteins, pET-28a-PDCoV-N plasmid was used as a template to amplify the target gene with primers containing *Eco*RI and *Xho*I restriction enzyme sites (Table 2). The obtained genes were digested and cloned into the prokaryotic expression vector pET-32a (+), which contained a His-tag, and then verified by sequence analysis. The different truncated fragments of the PDCoV N gene were expressed using the same method as the whole N protein expression.

### 2.5. Enrichment and Screening of scFv

For the enrichment and screening of positive scFvs, a 96-well ELISA plate was coated with 1 μg of the purified PDCoV N protein (100 μL/well) at 4 °C for 16 h and blocked with 5% non-fat milk (*w*/*v*) at 37 °C for 2 h. About 1 × 10^11^ pfu of the input phage was added to the well and incubated at 37 °C for 2 h. The plates were washed with PBS containing 0.05% tween-20 (PBST) several times (10 times for round 1 and 20 times for the other rounds). Specific antigen-bound phages were eluted with 100 μL of trypsin (1.75 ng/mL); then, the eluted phages were added into 3 mL *E. coli* XL1-Blue (OD_600_ = 0.6) and incubated at 37 °C for 1 h. From the mixture, 10 μL was gradient-diluted with PBS and coated on a 2 × YT-AG plate for titer calculation, while the remaining was incubated in 2 × YT-A medium and infected with M13KO7 to prepare the second phage antibody library. The above steps were repeated four times, and the input and output phage titers of each round were calculated and recorded.

### 2.6. Phage–Indirect Enzyme-Linked Immunosorbent Assay (ELISA)

After four rounds of screening, 54 colonies were randomly selected for identification by colony PCR using primers pSEX81-F and pSEX81-R. The antigen-binding activity of the positive clones was further identified by phage-ELISA. For small-scale phage preparation, the positive colonies were inoculated into 15 mL 2 × YT-A medium and cultured at 37 °C until OD_600_ = 0.6. The culture was infected with M13KO7 at 37 °C for 1 h. Bacteria were collected by centrifugation and resuspended in 30 mL of 2 × YT-AK medium. The bacteria culture was further incubated overnight at 30 °C with shaking at 260 rpm to produce phage particles. A 96-well ELISA plate was coated with 200 ng purified PDCoV N protein (100 μL/well) at 4 °C for 16 h, while PBST containing 1% (*w*/*v*) bovine serum albumin (BSA) was used as a negative control. Then, the wells were blocked with 5% non-fat milk at 37 °C for 2 h, and 1 × 10^8^ pfu of the freshly prepared phage was added and incubated at 37 °C for 2 h. The plate was washed with PBST five times, and the horseradish peroxidase (HRP)-conjugated anti-M13 antibody (Abcam, Cambridge, UK; 1:4000 dilution) was added as a secondary antibody. The wells were incubated at 37 °C for 1 h and washed with PBST five times. Then, 100 μL tetramethylbenzidine solution (Solarbio, Beijing, China) was added as chromogenic reagent, and 50 μL of 2M sulfuric acid solution was added to terminate the reaction. The absorbance at 450 nm was recorded by an ELISA reader (Gallop, Shanghai, China). The cut-off value was defined as a mean of OD from the negative control, plus threefold standard deviation (Cut-off  =  m  +  3SD).

### 2.7. Expression and Purification of scFv Antibodies

According to the phage-ELISA results, the colonies with higher affinity were sent for sequencing, and the results were analyzed using the IMGT website (https://www.imgt.org/IMGT_vquest/input; accessed on August 2019). The specific colony was incubated in 5 mL of 2 × YT-A medium, and the plasmid was extracted using the E.Z.N.A.TM plasmid Mini Kit (Omega, Norcross, GA, USA) according to the manufacturer’s instructions. Recombinant pSEX81-scFv plasmid and eukaryotic expression vector pFUSE-hIgG-Fc2 were simultaneously digested with *Sfi*I and ligated by T_4_ DNA ligase. The ligated product was transformed into *E. coli* DH5α competent cells and grown in low-salt LB medium containing 25 μg/mL zeocin (LS-LB-Z) at 37 °C for 16 h. The recombinant eukaryotic expression plasmid was extracted using an endo-free E.Z.N.A.TM plasmid DNA Mini Kit (Omega, Norcross, GA, USA) and then transfected to HEK-293T cells using the LipofectamineTM2000 (Invitrogen, Carlsbad, CA, USA). The transfected cells were incubated at 37 °C with 5% CO_2_ for 48 h. The culture supernatant and cell precipitate were collected to verify the expression of scFv-Fc by Western blot. The scFv-Fc antibody in the supernatant was purified using Protein A-sepharose (GE Healthcare Life Sciences, Pittsburgh, PA, USA) according to the manufacturer’s instructions. The purified antibody was analyzed using SDS-PAGE under the non-reducing condition (without the reducing agent dithiothreitol (DTT)) and the reducing condition (containing DTT), respectively.

### 2.8. Indirect ELISA

Indirect ELISA was used to identify the cross-reactivity between scFv-Fc and porcine diarrhea-related viruses (PDCoV, PEDV, TGEV and PSV). The ELISA plate was coated with UV-inactivated virus (100 μL/well, 10^6^ TCID_50_ per well) at 4 °C for 16 h, while the mock-infected ST and Vero cells were used as negative controls. The plates were blocked with 5% non-fat milk at 37 °C for 2 h, and the purified scFv-Fc (300 ng/well) was added and incubated at 37 °C for 2 h. The plate was washed with PBST five times, and the HRP-conjugated goat anti-human IgG (1:8000 dilution) was added as a secondary antibody. The plates were incubated at 37 °C for 1 h and washed with PBST five times. The absorbance at 450 nm was recorded by an ELISA reader.

### 2.9. Western Blot

Western blot was used to identify the activity of purified scFv-Fc binding to PDCoV. PDCoV (MOI = 1) was used to infect ST cells, and the cells were harvested at 24 h post-infection (hpi) and viral proteins were obtained from the cells using lysis RIPA buffer containing 10 mM phenylmethylsulfonyl fluoride (PMSF). The protein concentration was determined by BCA protein assay kit (Beyotime, Nantong, China). Then, the proteins were boiled with 6 × protein-loading buffer (TransGen, Beijing, China) and 40 μg samples were separated by 12% SDS-PAGE gel. The protein bands in the gel were transferred to a nitrocellulose filter (NC) membrane. The membranes were blocked with 5% non-fat milk at 37 °C for 2 h and further incubated with purified scFv-Fc (5 μg/mL) at 37 °C for 2 h. After being washed with PBST five times, HRP-conjugated goat anti-human IgG (Proteintech, Chicago, IL, USA; 1:4000 dilution) was added as the secondary antibody, and the membranes were incubated at 37 °C for 1 h. The NC membranes were washed five times with PBST and visualized with ECL reagents (Solarbio, Beijing, China). The final images were captured using an Amersham imager 680 (GE Healthcare Life Sciences, USA).

### 2.10. Indirect Immunofluorescence Assay

Indirect immunofluorescence assay (IFA) was performed to detect the reaction of the purified scFv-Fc antibody with PDCoV-infected cells. Briefly, ST cells were seeded onto a 12-well tissue culture plate (NEST, Wuxi, China) and grown to 90% confluent. Then, the cells were incubated with PDCoV (MOI = 1) at 37 °C with 5% CO_2_ for 1 h. After washing with PBS, the cells were incubated with maintenance medium (DMEM supplemented with 1% antibiotic-antimycotic and 1% pancreatin) until cytopathic effect (CPE) appeared. In general, CPE appeared about 12 hpi (MOI = 1). The cells were washed with PBST twice, fixed with 4% paraformaldehyde for 15 min and permeabilized with 0.5% Triton X-100 for 10 min. Then, the cells were blocked with 5% BSA for 1 h at 37 °C and incubated with 200 μL of purified scFv-Fc (30 μg/mL) for 12 h at 4 °C. After washing with PBST five times, the cells were incubated with 200 μL of fluorescein isothiocyanate (FITC)-conjugated goat anti-human IgG (Proteintech, Chicago, IL, USA; 1:400 dilution) for 1 h at 37 °C. Finally, the cells were washed with PBST five times and the nuclei were stained with 4’, 6-diamidino-2-phenylindole (DAPI, Solarbio, Beijing, China) for 10 min at room temperature. Fluorescence was observed with a fluorescence microscope (Olympus, Tokyo, Japan).

### 2.11. Dot Blot

The epitope on the PDCoV N protein recognized by the scFv-Fc was identified by dot blot. For antigen processing, bacteria containing recombinant plasmids of different truncated PDCoV N genes were induced and further resuspended in PBS. After boiling for 5 min, the supernatant was obtained by centrifugation. The NC membrane was spotted with 6 μL of supernatant, and the spotted samples were air-dried for 20 min and blocked with 5% non-fat milk at 37 °C for 1 h. For confirmation of the expression of the truncated PDCoV N protein, the NC membranes were incubated with HRP-conjugated mouse anti-His-tag antibodies (Bersee, Beijing, China; 1:4000 dilution). For identification of the epitope, the NC membrane was incubated with purified scFv-Fc antibody (5 μg/mL), followed by HRP-conjugated goat anti-human IgG (1:4000 dilution). The blots were visualized with ECL reagents and imaged using an Amersham imager 680.

### 2.12. Protein Modeling and Molecular Docking

Due to no available information about the three-dimensional structures of the PDCoV N protein and the selected scFv in the Protein Data Bank (PDB, https://www.rcsb.org/pdb/; accessed on 22 February 2022), we generated the three-dimensional structures of the PDCoV N protein and the scFv using SWISS-MODEL (swissmodel.expasy.org), a fully automated protein structure homology-modeling server. The predicted structures were displayed with PyMol software (PyMOL 1.7.6).

Molecular docking was performed to investigate the binding mode between the PDCoV N protein and the scFv using the ZDOCK server (https://zdock.umassmed.edu; accessed on 22 February 2022). For docking, the residues of the identified antigenic epitope of the PDCoV N protein were selected for the binding sites. The default parameters were used as described in the ZDOCK server if it is not mentioned. The top-ranked complex as judged by the ZDOCK score was subject to visually analyses using PyMoL.

## 3. Results

### 3.1. Construction of Phage Display scFv Library

**Figure 1:**
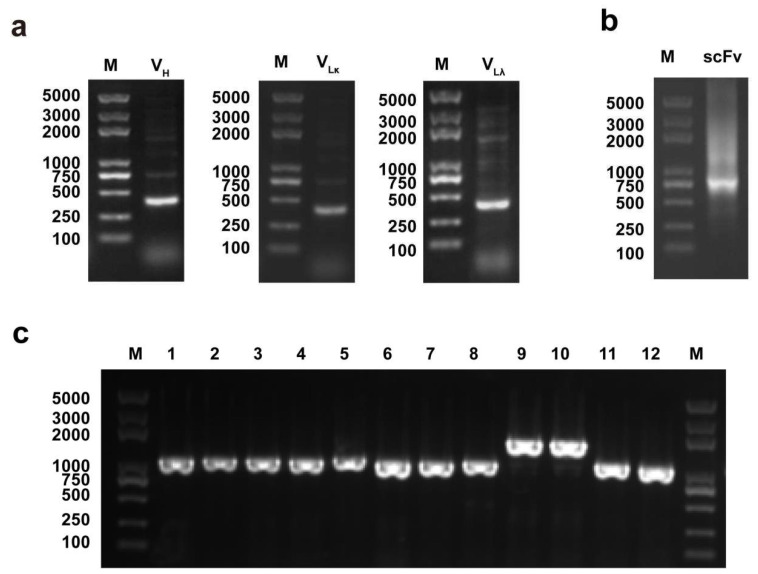
Identification of PCR products of scFv genes: (**a**) Amplification of V_H_ and V_L_ genes from the spleen tissue of PDCoV-infected piglets. Lane M, DL 5000 DNA marker; V_H_, PCR products of V_H_ gene; V_Lκ_, PCR products of V_Lκ_ gene; V_Lλ_, PCR products of V_Lλ_ gene. (**b**) Amplification of scFv by SOE-PCR. Lane M, DL 5000 DNA marker; scFv, assembled scFv gene. (**c**) Identification of primary antibody library. Lane M, DL 5000 DNA Marker; Lane 1–12, PCR amplification of the randomly selected colonies from scFv library.

The V_H_ and V_L_ fragments were amplified from the spleens of PDCoV-positive swine by PCR (Figure 1a). After purification, the V_H_ and V_L_ fragments were assembled into scFvs fragments by SOE-PCR. The full-length scFv gene was about 750 bp (Figure 1b). The scFv genes were further cloned into pSEX-81 phagemid vector and electroporated into competent *E. coli* XL1-Blue. After incubation for 16 h at 37 °C, 12 colonies were randomly selected to identify the inserted scFv by colony PCR. The correct size should be about 1100 bp, and 10 out of the 12 colonies were correctly confirmed by gene sequencing (Figure 1c), which indicated that the proportion of recombinant plasmids containing scFv was about 83%. The storage capacity of the primary antibody library was about 1.7 × 10^8^ cfu.

### 3.2. Expression and Purification of PDCoV N Protein

**Figure 2:**
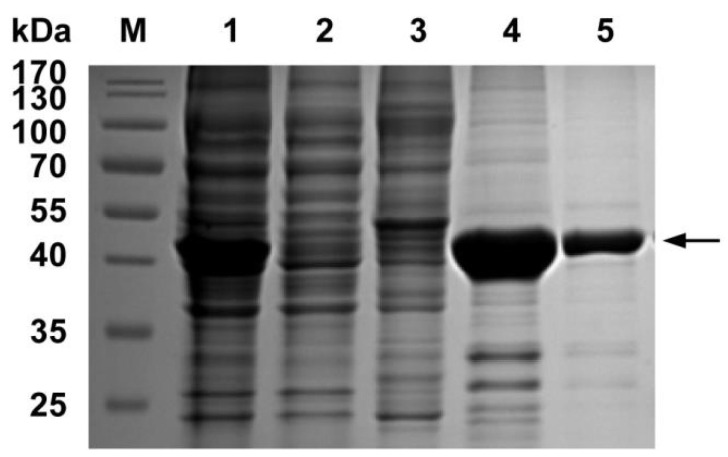
Expression and purification of the PDCoV N protein. Lane M, protein marker; Lane 1, induced *E. coli* BL21 (DE3) with pET-28a-PDCoV-N; Lane 2, induced *E. coli* BL21 (DE3) with empty vector pET-28a; Lane 3, precipitation of the PDCoV N protein strain after ultrasonication; Lane 4, supernatant of the PDCoV N protein strain after ultrasonication; Lane 5, purified PDCoV N protein.

The recombinant plasmid pET-28a-N was constructed and transformed into competent *E. coli* BL21 (DE3). The expression of the PDCoV N protein was induced by 0.8 mM IPTG at 37 °C for 5 h and purified by nickel affinity chromatography. The SDS-PAGE analysis showed that the PDCoV N protein was mainly expressed in supernatant with a predicted molecule weight of 45 kDa. The concentration of the purified PDCoV N protein was 1.6 mg/mL. The purified protein was verified by SDS-PAGE, as shown in Figure 2.

### 3.3. Screening and Identification of scFv against PDCoV N Protein

**Figure 3:**
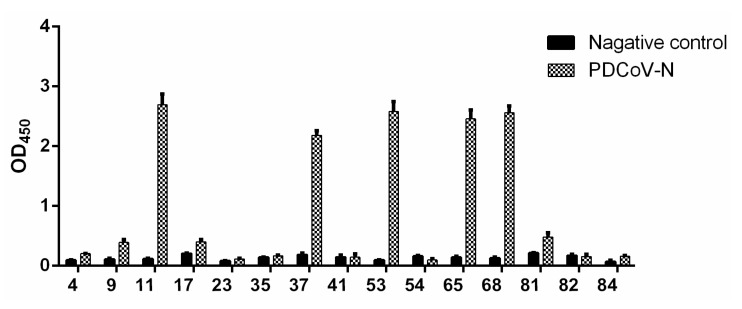
Detection of the reactivity of selected scFvs with the PDCoV N protein. As negative control, 1% BSA was used. The plates were coated with purified PDCoV N protein and incubated with selected phages, followed by HRP-conjugated anti-M13 antibody for visualization. Date represent the average values of three independent assays.

**Figure 4:**
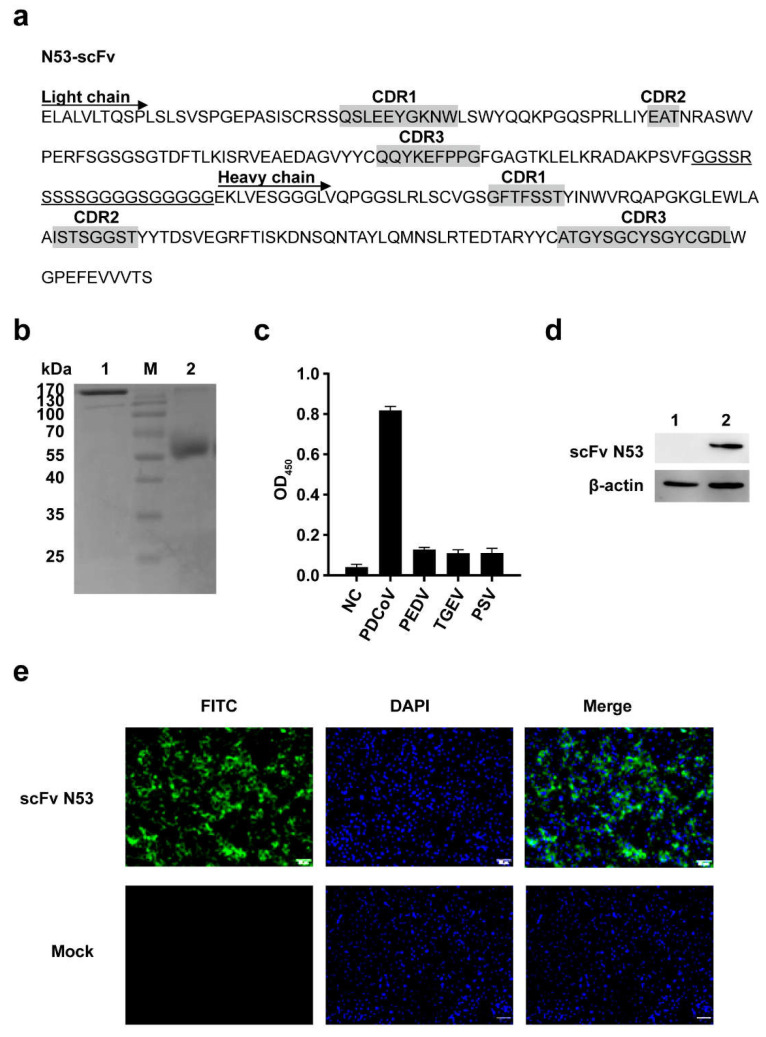
Expression, purification and identification of scFv N53: (**a**) The scFv sequence of colony 53 was sequenced, and the complementary determining regions (CDR) were identified using IMGT/VQUEST. (**b**) SDS-PAGE analysis of the purified scFv N53. The scFv sequence of colony 53 was cloned into pFUSE-hIgG-Fc2 vector, and scFv N53 was expressed in HEK-293T cells. Lane M, protein marker; Lane 1, 2 μg purified scFv N53 under non-reducing condition (-DTT); Lane 2, 2 μg purified scFv N53 under reducing condition (+DTT). (**c**) Specificity of scFv N53 was determined by ELISA. Plates that were coated with PDCoV, PEDV, TGEV and PSV were incubated with scFv N53, followed by HRP-conjugated anti-M13 antibody. Data represent the average values of three independent assays. (**d**) The reactivity of scFv N53 with the PDCoV N protein was analyzed by Western blot. Lane 1, mock-infected ST cells; Lane 2, PDCoV-infected ST cells. (**e**) Reactivity of scFv N53 with PDCoV in ST cells was determined by IFA. PDCoV-infected or mock-infected ST cells were incubated with scFv N53, followed by FITC-conjugated goat anti-human IgG (1:400 dilution). DAPI was used to visualize the cell nuclei. Cells were observed under fluorescence microscope (Maganification: 200×).

The phage display scFv library was screened by four rounds of adsorption–elution–enrichment using ELISA with the purified PDCoV N protein. The input and output phage titer of each round and the output/input ratio are listed in Table 3. The harvest rate of phage antibodies was increased from 8.4 × 10^−6^ to 2.6 × 10^−3^, which indicated that the specific phage antibodies were enriched about 310-fold. After four rounds of enrichment, 54 colonies were randomly selected for identification by colony PCR, and 15 colonies were confirmed with a correct size as scFv. The binding activity of positive colonies was determined by phage-ELISA. The results showed that five colonies (11, 37, 53, 65, 68) had a high affinity to the PDCoV N protein (Figure 3). Then, the five clones were sequenced and further analyzed using IMGT/V-QUEST. The results showed that the sequenced scFv had a variable region structure and gene characteristics (Figure 4a).

### 3.4. Expression and Purification of scFv-Fc

The scFv gene (colony 53) was cloned into pFUSE-hIgG-Fc2. The recombinant plasmid was transiently transfected to HEK-293T cells in a 6-well plate and incubated for 48 h at 37 °C with 5% CO_2_. The expressed scFv N53 in supernatant was purified by Protein A-sepharose. The SDS-PAGE analysis showed the expressed scFv N53 was about 170 kDa under the non-reducing condition and ~55 kDa under the reducing condition (Figure 4b), indicating that the scFv-Fc polymer was formed as expected.

### 3.5. Analysis of the Specificity and Affinity of the Purified scFv N53

Indirect ELISA, Western blot and IFA were performed to identify the specificity and affinity of the purified scFv N53. Indirect ELISA showed the purified scFv-Fc antibody only reacted with PDCoV but not with PEDV, TGEV or PSV (Figure 4c). Western blot analysis showed that scFv N53 was able to recognize the PDCoV N protein (Figure 4d). The results of IFA also showed that the purified scFv N53 could react with PDCoV-infected cells (Figure 4e). The above results suggested that the purified scFv N53 had good specificity and reactivity with PDCoV.

### 3.6. Identification of Epitope Recognized by the scFv N53

**Figure 5:**
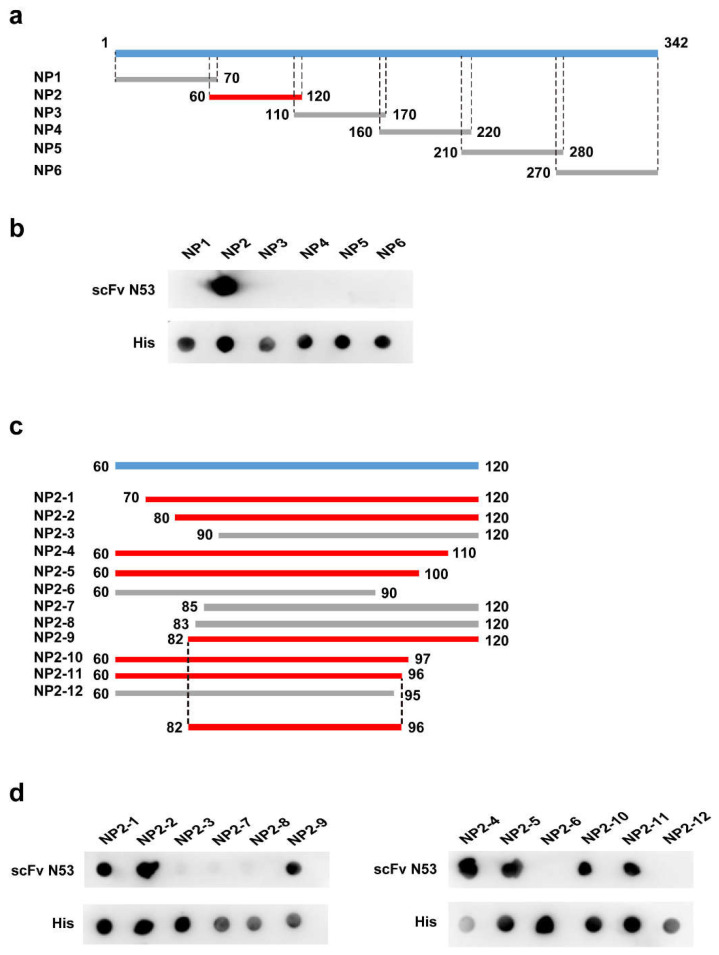
Mapping of PDCoV N protein epitopes: (**a**,**c**) Schematic diagram of the epitope mapping. The blue bands represent the whole PDCoV N protein (**a**) and the fragment NP2 (**b**). The segments that can be recognized by scFv N53 are highlighted in red, and the segments that cannot react with scFv N53 are marked in gray. (**b**,**d**) Binding activity of scFv N53 to various truncated N proteins was determined by dot blot. The proteins were expressed and spotted on NC membrane, then incubated with scFv N53 and anti-His antibody, respectively.

To identify the epitope on the PDCoV N protein recognized by scFv N53, six mutually overlapping fragments (Figure 5a) covering the complete PDCoV N gene with approximately equal length were expressed in *E. coli* BL21 (DE3) with His-tag (NP1, NP2, NP3, NP4, NP5, NP6). Then, the reactivity of the truncated proteins with scFv N53 was confirmed by dot blot, respectively. As shown in Figure 5b, the purified scFv N53 only bound to the NP2 protein. To locate the precise position of the epitope, the NP2 was further truncated, as depicted in Figure 5c, and expressed in *E. coli* BL21 (DE3), which was detected by dot blot assay. The results (Figure 5d) indicated that 82GELPPNDTPATTRVT96 was the precise epitope recognized by scFv N53.

### 3.7. Docking Analysis of scFv and N Protein Interactions

**Figure 6:**
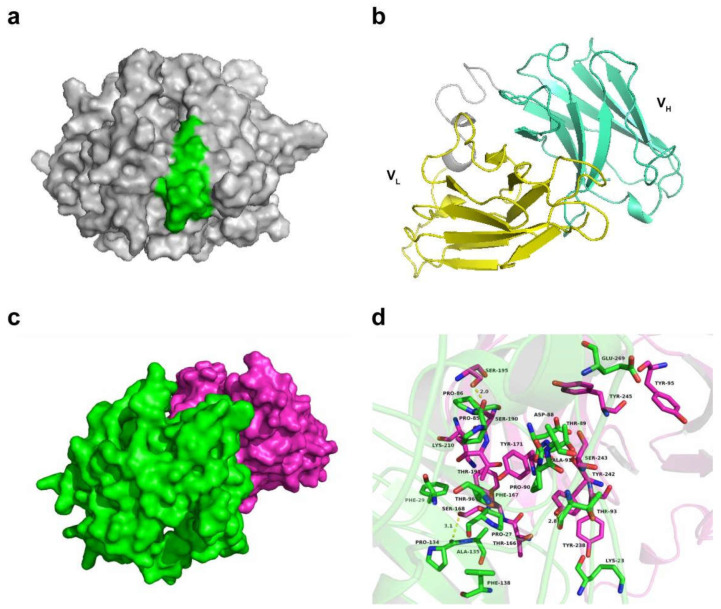
The binding site of the PDCoV N protein and scFv N53. (**a**) Predicted structure model of the PDCoV N protein. The epitope recognized by scFv N53 is marked in green. (**b**) Predicted structure model of scFv N53. The V_L_ region is marked in yellow and the V_H_ region is marked in light blue. (**c**) Total view of the interaction between the PDCoV N protein (green surface mode) and scFv N53 (rose red surface mode). (**d**) Detailed view of the interaction between the PDCoV N protein (green) and scFv N53 (rose red). The representative residues are shown in the green sticks for the PDCoV N protein and the rose red sticks for scFv N53. The hydrogen bond is displayed in yellow dotted line.

Since the antigen-binding site is located in a variable region, we removed the Fc region of scFv N53 in modeling and analysis. The structure homology modeling of the PDCoV N protein and scFv N53 were constructed using SWISS-MODEL. The predicted structures were displayed with PyMoL (Figure 6a,b). The interactions between the PDCoV N protein (green) and scFv N53 (rose red) are shown in Figure 6c. A detailed analysis showed that a hydrophobic interaction was observed between the residues Pro-27, Phe-29 and Pro-134 of the PDCoV N protein and residue Phe-167 of scFv N53 (Figure 6d). In addition, the residues Lys-23 and Phe-29 of the PDCoV N protein formed cation-π interactions with the residues Tyr-238 and Lys-210 of scFv N53, respectively. The residues Asp-88 and Glu-269 of the PDCoV N protein formed anion-π interactions with the residues Tyr-245 and Tyr-95 of scFv N53, respectively. Importantly, three hydrogen-bond interactions were shown between the residue Pro-85 of the PDCoV N protein and the residue Ser-195 of scFv N53 (bond length: 2.0 Å), the residue Thr-93 of the N protein and the residue Tyr-242 of scFv N53 (bond length: 2.8 Å), and the residue Pro-134 of the N protein and the residue Ser-168 of scFv N53 (bond length: 3.1 Å), which represented the main binding affinity between the PDCoV N protein and scFv N53 (Figure 6d). 

## 4. Discussion

The potentially broad host range of PDCoV has posed a major threat to human health. PDCoV mainly infects pigs, but it can also infect chickens and calves with cross-species transmission in laboratory conditions [22,23]. At present, there is no specific therapy reagent for PDCoV infection. The N protein is abundantly expressed in cells after coronaviral infection and plays a key role in the early stages of virus infection [24,25]. Because the N protein is highly conserved and less prone to mutation, it is quite suitable for virus diagnosis, and it also plays an important role in basic research on the pathogenesis and immune mechanism of PDCoV.

In this study, we obtained a specific scFv against the PDCoV N protein using phage display technology, and the antigenic epitopes of the PDCoV N protein were further confirmed. First, a porcine phage scFv library was constructed from the spleen cells of PDCoV-infected diarrhea piglets. The storage capacity of the primary antibody library reached 1.7 × 10^8^ cfu, and the proportion of recombinant plasmids containing scFv was about 83%, indicating that antibodies against PDCoV proteins are able to be screened. The scFv gene was spliced into the heavy-chain variable region and light-chain variable region, connected with a flexible linker. Studies have shown that the affinity of V_H_-linker-V_L_ formation is ten times higher than V_L_-linker-V_H_ formation, while the expression level of V_L_-linker-V_H_ is twenty times higher than V_H_-linker-V_L_ formation [26]. For ensuring the expression level and improving the affinity of scFv-Fc, VL-linker-VH was chosen. The specific phage scFvs against the PDCoV N protein were enriched by four rounds of biopanning. The sequencing results showed that the phage scFvs with a high affinity to the N protein were all consistent, indicating that the specific scFvs were successfully enriched.

The selected scFv is expected to be actively expressed. As the prokaryotic expression system has the advantage of a short expression cycle, a large amount of production and a low cost, it is widely used [27]. In the current study, the selected scFv gene in *E. coli* host was chosen to express. However, the scFv was found to express in the form of an inclusion body when using pET28a or pET32a plasmid and showed no activity after re-folding. Then, the eukaryotic expression system with pFUSE plasmid containing human Fc fragment was used. The biggest drawback of scFv is its monovalent binding to the target antigen. Even though its monovalent binding has a high affinity, the dissociation occurs quickly, which makes it unable to stay at the target site for a long time in a non-equilibrium environment in vivo. The binding of scFv to the Fc fragment using the recombinant fusion method not only prolongs the half-life of scFv-Fc in vivo, but also facilitates the purification of the antibody. According to previous research, the expression form of scFv-Fc can also significantly improve the antibody’s affinity [15,28,29]. Thus, the selected scFv was conducted by recombinant pFUSE-hIgG-Fc2 plasmid and transfected to HEK-293T cells. The purified antibody was obtained from the culture supernatant. According to the results of indirect ELISA, Western blot and IFA, scFv N53 had good specificity and high reactivity with the PDCoV N protein and PDCoV-infected cells.

Then, we clarified the location of the antigenic epitope on the PDCoV N protein recognized by scFv N53 using dot blot analysis. The precise epitope recognized by scFv N53 was 82GELPPNDTPATTRVT96. To further clarify the interaction mechanism of scFv N53 with the PDCoV N protein, we predicted the 3D structure of the PDCoV N protein and scFv N53, respectively, and used ZDOCK to analyze the interaction site. The hydrogen-bonding interactions between the residues Pro-85, Thr-93 and Pro-134 of the PDCoV N protein and the residues Ser-195, Tyr-242 and Ser-168 of scFv N53 determined the primary binding affinity of the PDCoV N protein to scFv N53. Interestingly, the residues Pro-85 and Thr-93 were located on the antigenic epitope we identified, while residue Pro-134 was not. Further analysis found that the bonding energies of hydrogen bonds formed by residues Pro-85 and Thr-93 were both greater than that of residue Pro-134, so we speculated that residues Pro-85 and Thr-93 of the PDCoV N protein play critical roles in spatial conformation. Overall, the above molecular simulations give us a rational explanation of the interaction between the PDCoV N protein and scFv N53, and the specific interaction mechanism needs to be further explored.

In conclusion, we screened and characterized an scFv-Fc antibody (scFv N53) against the PDCoV N protein by phage display technology. ScFv N53 exhibited high sensitivity and specificity in antibody–antigen reactions. The antigenic epitope was identified, and the interaction mode of scFv with the PDCoV N protein was analyzed. These results suggest that scFv N53 can be used as an effective tool for the research of the PDCoV N protein and the development of a diagnosis reagent for PDCoV.

## Figures and Tables

**Table 1:** Primers for porcine antibody variable fragments.

Primer	Sequence(5’−3’)
VH-1F	GGTGGTTCCTCTAGATCTTCCTCCTCTGGTGGCGGTGGCTCGGGCGGTGGTGGGGAGGWGAAGCTGGTGGAGTCYGG
VH-2F	GGTGGTTCCTCTAGATCTTCCTCCTCTGGTGGCGGTGGCTCGGGCGGTGGTGGGSAGGTSCAGCTGGTRCAGTCTGG
VH-3F	GGTGGTTCCTCTAGATCTTCCTCCTCTGGTGGCGGTGGCTCGGGCGGTGGTGGGSAGGTGCAGCTGKTGGAG
VH-1R	CCTGGCCGGCCTGGCCACTAGTCACGACGACTTCAACGCCTGG
VH-2R	CCTGGCCGGCCTGGCCACTAGTCACGACGACTTCRAYGCCTGG
VH-3R	CCTGGCCGGCCTGGCCACTAGTCACGACGACTTCRACKCCTGG
VLκ-1F	GGGCCCAGGCGGCCGAGCTCGCCMTYGTGCTGACCCAGTCTCCA
VLκ-2F	GGGCCCAGGCGGCCGAGCTCGAGMTCGTSATGACCCAGTCTCCA
VLκ-3F	GGGCCCAGGCGGCCGAGCTCGMCATCCRGWTGACCCAGTCTCCA
VLκ-1R	GGAAGATCTAGAGGAACCACCTTTGAKYTCCAGCTTGGTCCC
VLκ-2R	GGAAGATCTAGAGGAACCACCTTTGATATCCACTTTGGTCCC
VL_λ_-1F	GGGCCCAGGCGGCCGAGCTCTCTTCTAAGCTGACTCAGCCCCCGGGGGTGT
VL_λ_-1R	GGAAGATCTAGAGGAACCACCCCGTGGGAGYGGCCTTGGGCTGACC
scFv-F	GAGGAGGAGGAGGAGGAGGCGGGGCCCAGGCGGCCGAGCTC
scFv-R	GAGGAGGAGGAGGAGGAGCCTGGCCGGCCTGGCCACTAGT

**Table 2:** Primers used for the complete and truncated N genes of PDCoV.

Primers	Sequences(5′–3′)	Amino Acid Position
PDCoV N	(F) CG*GAATTC*ATGGCCGCACCAGTAGTCC	1–342
	(R) CC*CTCGAG*CGCTGCTGATTCCTGCTTTA	
NP-1	(F) CG*GAATTC*ATGGCCGCACCAGTAGTCC (R) CCG*CTCGAG*ATAATAAAAGGCATAGGATGGAGGA	1–70
NP-2	(F) CG*GAATTC*ACTCCGATTCCTCCATCCTATGCCT (R) CCG*CTCGAG*TTTAGGATTGTTGGGGTTGCGTTTG	60–120
NP-3	(F) CG*GAATTC*CATGTTGCCAAACGCAACCC (R) CCG*CTCGAG*GGGCTGATTGCCTGTGCCTCT	110–170
NP-4	(F) CG*GAATTC*TCTGTTAACTCCAGAGGCACAGG (R) CCG*CTCGAG*CTCAGTGTCTGCAGAGCCGACAT	160–220
NP-5	(F) CG*GAATTC*ACTGGTGCCAATGTCGGCT (R) CCG*CTCGAG*CGCATCCTTAAGTCTCTCATAGTCA	210–280
NP-6	(F) CG*GAATTC*GGTTCTCCTGACTATGAGAGACTTA (R) CCG*CTCGAG*CGCTGGTGATTCCTGCTTTATCTCA	270–342
NP-2-1	(F) CG*GAATTC*TATACTGGCACAGGTCCCAGAGGAA (R) CCG*CTCGAG*TTTAGGATTGTTGGGGTTGCGTTTG	70–120
NP-2-2	(F)CG*GAATTC*AAGTATGGTGAACTCCCTCCTAATG (R) CCG*CTCGAG*TTTAGGATTGTTGGGGTTGCGTTTG	80–120
NP-2-3	(F) CG*GAATTC*CCAGCAACCACTCGTGTTACTTGG (R) CCG*CTCGAG*TTTAGGATTGTTGGGGTTGCGTTTG	90–120
NP-2-4	(F) CG*GAATTC*ACTCCGATTCCTCCATCCTATGCCT (R) CCG*CTCGAG*ATGAGGTTTAATAGAAGTGTCAGCT	60–110
NP-2-5	(F) CG*GAATTC*ACTCCGATTCCTCCATCCTATGCCT (R) CCG*CTCGAG*ACCCTTAACCCAAGTAACACGAGTG	60–100
NP-2-6	(F) CG*GAATTC*ACTCCGATTCCTCCATCCTATGCCT (R) CCG*CTCGAG*TGGGGTATCATTAGGAGGGAGTTCA	60–90
NP-2-7	(F) CG*GAATTC*TATGGTGAACTCCCTCCTAATGATA (R) CCG*CTCGAG*TTTAGGATTGTTGGGGTTGCGTTTG	81–120
NP-2-8	(F) CG*GAATTC*GGTGAACTCCCTCCTAATGATACCC (R) CCG*CTCGAG*TTTAGGATTGTTGGGGTTGCGTTTG	82–120
NP-2-9	(F) CG*GAATTC*GAACTCCCTCCTAATGATACCCCAG (R) CCG*CTCGAG*TTTAGGATTGTTGGGGTTGCGTTTG	83–120
NP-2-10	(F) CG*GAATTC*ACTCCGATTCCTCCATCCTATGCCT (R) CCG*CTCGAG*AACACGAGTGGTTGCTGGGGTAT	60–95
NP-2-11	(F) CG*GAATTC*ACTCCGATTCCTCCATCCTATGCCT (R) CCG*CTCGAG*AGTAACACGAGTGGTTGCTGGGGTA	60–96
NP-2-12	(F) CG*GAATTC*ACTCCGATTCCTCCATCCTATGCCT (R) CCG*CTCGAG*CCAAGTAACACGAGTGGTTGCTG	60–97

Note: Location of the fragments encoded by these primers is based on the sequence of the N gene of PDCoV strain CH-01 (GenBank accession number: KX443143.2).

**Table 3:** The phage library titer input and output numbers of each panning.

Round of Screening	Input Number of Phage (pfu)	Output Number of Phage (pfu)	Output/Input
1	4.5 × 10^9^	3.8 × 10^4^	8.4 × 10^−6^
2	1.2 × 10^10^	3 × 10^6^	2.5 × 10^−4^
3	3.9 × 10^9^	8.7 × 10^6^	2.2 × 10^−3^
4	1.7 × 10^10^	4.5 × 10^7^	2.6 × 10^−3^

## Data Availability

The authors promise the availability of all the data and materials that are presented in the manuscript.
